# Priapism as the first manifestation of chronic myeloid leukemia

**DOI:** 10.4103/0256-4947.55176

**Published:** 2009

**Authors:** Ilias Tazi

**Affiliations:** From the Department of Medicine, Hematology Unit, Centre Hospitalier Regional, Beni-Mellal, Morocco

**To The Editor:** A previously healthy 33-year-old Moroccan man was referred to the local hospital for persistent painful erection of the penis that had lasted approximately 22 hours. His penis remained erect, painful, and swollen when he arrived at the emergency department. He denied recent intercourse, trauma, use of illicit drugs, use of medications, and radiation therapy. The patient also denied fever, sweats, and chills. The vital signs revealed a body temperature of 37.4 °C, pulse of 98 beats/minute, and blood pressure of 12/7 mm Hg, and respiration of 22 beats/minute. He was alert and oriented. The physical examination revealed that the spleen was palpable 4 cm below the left costal margin. His conjunctiva was pale but no jaundice. On examination, the penis was erect, firm, and tender with superficial venous engorgement. Urinalysis was normal. Laboratory data showed hemoglobin 10.53 g/dL, hematocrit 24%, white blood count 400000/mm^3^, and platelets 1200000/mm^3^. A peripheral blood smear demonstrated immature leukocytes in various stages of differentiation. Karyotype analysis revealed the Ph1 chromosome and myeloid hyperplasia in the bone marrow. A diagnosis of chronic myeloid leukemia (CML) was made. Treatment of the priapism was initially performed by cavernosa aspiration and epinephrine irrigation at emergency department under the impression of low flow-type priapism. The erection was relieved later by these procedures. For hyperleukocytosis, the patient was admitted to the hematological ward. He was started on hydroxyurea therapy (50 mg/kg/day) associated with intravenous fluid hydration and allopurinol (300 mg/day) for potential tumor lysis syndrome. After 15 days of chemotherapy with hydroxyurea, the patient's leukocyte and platelet counts were 8000/mm^3^ and 240000/mm^3^, respectively. Recurrent priapism had not happened to him during his admission period. The patient was treated with imatinib mesylate for his CML and continued to report to us without any erectile dysfunction until the date of writing.

Priapism is a rare disease, characterized by prolonged, painful and irreducible erection, not resulting in ejaculation. It is an andrological emergency with a poor prognosis, as the risk of impotence is 50% despite appropriate management. Sickle cell anaemia, chronic myelogenous leukemia, chronic lymphocytic leukemia, and acute lymphoblastic leukemia are hematologic disorders that can be a cause of priapism.^1^ In adult leukemic patients, the incidence of priapism is estimated to be approximately 5%.^2^ Priapism as a result of hematologic malignancy is most likely caused by venous obstruction from microemboli/thrombi as well as hyperviscosity caused by the increased number of circulating leukocytes in mature and immature forms.^3^ In cases of hematologic malignancy, controversy has existed regarding the optimal treatment of leukemic priapism. Earlier series of case reports show successful detumescence with local radiation therapy, open surgical shunting, or combination of the two treatments.^4^ More recent literature has focused on the use of cytoreductive modalities such as chemotherapy or combination chemotherapy and leukapheresis.^5^ Because of the relatively rare occurrence of leukemic priapism and the small number of case series, there is no standard treatment protocol. Chemotherapy or radiotherapy may first be attempted. If detumescence is not achieved, then surgical shunting should be considered.
